# Enhanced photoluminescence of porous silicon nanoparticles coated by bioresorbable polymers

**DOI:** 10.1186/1556-276X-7-446

**Published:** 2012-08-08

**Authors:** Maxim B Gongalsky, Alexander Yu Kharin, Liubov A Osminkina, Victor Yu Timoshenko, Jinyoung Jeong, Han Lee, Bong Hyun Chung

**Affiliations:** 1 Physics Department, Lomonosov Moscow State University, Leninskie Gory 1, Moscow, 119991, Russia; 2 Major in Nanobioengineering, University of Science and Technology, Daejeon, 305-806, South Korea; 3 BioNanotechnology Research Center, Korea Research Institute of Bioscience and Biotechnology, 125 Gwahangno Yuseong, Daejeon, 305-806, South Korea

**Keywords:** Silicon nanoparticles, Porous silicon, Bioimaging, Polymer coating, Photoluminescence

## Abstract

A significant enhancement of the photoluminescence (PL) efficiency is observed for aqueous suspensions of porous silicon nanoparticles (PSiNPs) coated by bioresorbable polymers, i.e., polylactic-co-glycolic acid (PLGA) and polyvinyl alcohol (PVA). PSiNPs with average size about 100 nm prepared by mechanical grinding of electrochemically etched porous silicon were dispersed in water to prepare the stable suspension. The inner hydrophobic PLGA layer prevents the PSiNPs from the dissolution in water, while the outer PVA layer makes the PSiNPs hydrophilic. The PL quantum yield of PLGA/PVA-coated PSiNPs was found to increase by three times for 2 weeks of the storage in water. The observed effect is explained by taking into account both suppression of the dissolution of PSiNPs in water and a process of the passivation of nonradiative defects in PSiNPs. The obtained results are interesting in view of the potential applications of PSiNPs in bioimaging.

## Background

Optical techniques such as luminescent labeling are widely used in biomedicine today. They are noninvasive and can be employed for *in vitro* and *in vivo* diagnostics. One example is *in vitro* tests on infectious diseases based on a photoluminescence (PL) response, e.g., Gram staining [1]. Another example is the optical coherent tomography, which is successfully employed to detect malignant tumors *in vivo*[2]. In this case the cost of a single analysis is several times lower than that of radiology treatment. The next field of optical diagnostics is the fluorescent labeling of antibodies in order to estimate efficiency of targeted chemotherapy *in vivo*[3]. The dendrite cells were tracked by combining the fluorescent labeling with magnetic resonant imaging [4]. In the latter work, fluorescent indocyanine green and magnetic iron oxides embedded into polylactic-co-glycolic acid (PLGA) nanoparticles were used for high resolution diagnostics of lymph nodes. The employed PLGA is a well-known biocompatible polymer for drug delivery applications [5].

Porous silicon (PSi) is known to be potentially applicable in biomedicine [6]. Luminescent PSi consists of a network of silicon nanocrystals (nc-Si) with typical sizes of 2 to 5 nm separated by nanometer-sized pores [7]. The origin of PL is assumed to be the radiative recombination of charge carriers, i.e., electrons and holes coupled in excitons in nc-Si [8]. The quantum confinement for change carriers in nc-Si leads to a significant rise of the PL intensity and spectral shift. Thus, despite that the band gap in bulk crystalline silicon (c-Si) corresponds to the emission wavelength lying in the infrared spectral range, nc-Si can emit PL in the visible range [6,7]. The PL quantum yield of individual nc-Si could be as high as 60% [9]. Note that nc-Si are not only potential PL labels, but they can also be used for photodynamic therapy [10-12].

Quantum dots like CdSe or ZnS could also be used for bioimaging applications [13], but their cytotoxicity is rather high in comparison to nc-Si [14]. Long term biocompatibility is also present due to bioresorbable properties of nc-Si. The dissolution rate of nc-Si in aqueous solution depends on the pH level (acidity or alkalinity) and varies from 1 nm/day to 1 μm/day [15].

There are some reports about successful bioimaging by nc-Si both *in vitro*[16] and *in vivo*[17]. The PL properties of colloidal Si-based nanoparticles were demonstrated to exhibit degradation versus time because of dissolution in water [16]. In order to prevent this effect, one needs to use a specific surface coverage. First of all, this coverage should be bioresorbable in order to maintain the bioresorbability of the whole nanoparticles. Secondly, the coverage should protect nc-Si from agglomeration and should stabilize nc-Si-based suspension.

For example, in [18] authors report on *in vivo* imaging of sentinel lymph nodes by using luminescent nc-Si obtained by laser decomposition of silane. They used carboxylation of nc-Si surface followed by conjugation with specific biomolecules. The prepared nc-Si possessed efficient PL for several hours. Note that the carboxylation does not drastically change the PL intensity and spectral shape inherent for nc-Si. Another case is amine- or methyl-terminated nc-Si described in [19,20]. The organic groups that are covalently bound to the silicon surface induce a blue shift of the PL peak position. Furthermore, the amine- or methyl-terminated nc-Si is characterized by PL lifetimes in the range of nanoseconds, which are significantly shorter than that for uncovered nc-Si. Authors in [18,19] also reported about a significant increase of the PL quantum yield. These results indicate the great potential of an organic coverage for the modification of PL properties of nc-Si. Another interesting approach is based on microplasma treatment of PSi nanoparticles (PSiNPs) in a mixture of water and ethanol [21]. Free radicals created by the plasma decomposition of ethanol molecules lead to alcoxide-based coating of PSiNPs, and the formed coating stabilized the PL properties of PSiNPs. In [22] H-terminated nc-Si, obtained by partial dissolution of thermally treated silicon suboxide, were covered by solid lipids and were used for labeling of human breast cells *in vitro*. While the structural properties of the prepared nanoparticles were well-controlled, their PL intensity was rather low. Similar results have been recently demonstrated by using uncoated PSiNPs [23].

The present paper is aimed to study PSiNPs coated by biocompatible and biodegradable polymers as PLGA and polyvinyl alcohol (PVA) for bioimaging application. The PLGA/PVA compound is well-known amphiphilic coverage, because PVA strongly binds to PLGA by van der Waals forces [24]. Our results demonstrate that PLGA/PVA-coated PSiNPs possess the efficient PL for longtime storage in water.

## Methods

PSi films were prepared by electrochemical etching of boron-doped (100) c-Si wafers (specific resistivity of 1…10 Ohm*cm) in a mixture of HF (48%):C_2_H_5_OH (1:1) under etching current density 60 mA/cm^2^ for 40 min. The etching was done in a Teflon cell with a platinum counter electrode at room temperature.

In order to obtain free-standing PSi films, a short pulse of the etching current approximately 600 mA/cm^2^ was applied. The free-standing films were rinsed in deionized water and dried in air.

The porosity of the films was measured about 60% ± 5% by using the gravimetric analysis and low temperature nitrogen adsorption Brunauer-Emmet-Teller (BET) method. The BET method allowed us to estimate the mean diameter of pores equal to 4 ± 1 nm.

The dried films were hand-milled in agate mallet for 15 min to get powder. The prepared powder consisted of small individual PSiNPs (sizes of 10 to 200 nm) and larger particles (sizes above 200 nm) detected by using transmission electron microscopy (not shown).

The prepared powder was covered by PLGA/PVA in the following way. At the first step, the powder was mixed with dimethyl sulfoxide (DMSO) to obtain a suspension with particle concentration approximately 1 mg/ml. Then the suspension was centrifuged for 3 min at 2,000 rpm (rotor's radius approximately 40 cm) in order to remove largest particles. The supernatant was ultrasonicated for 4 h in order to form the stable suspension. At the second step, 1 ml of the suspension was mixed with 40 mg of PLGA, and the mixture was stirred for 1 h. Poly(d,l-lactide-co-glycolide) (PLGA, MW:5, 000 Da) was purchased from Boehringer Ingelheim Inc (Ridgefield, CT, USA). Polyvinyl alcohol (PVA, MW:20,000 Da) and DMSO were obtained from Sigma-Aldrich Corporation (St. Louis, MO, USA).

In the third step, the suspension of PLGA-coated PSiNPs (1 ml) was mixed with 9 ml of aqueous solution of PVA (45 mg/ml). Then the suspension of PLGA/PVA-coated PSiNPs was stirred for 20 h in order to create a hydrophilic coverage of PSiNPs and to prevent their agglomeration. At the final step, the nanoparticles were triply precipitated by centrifugation (3,000 rpm, 15 min) followed by washing and stirring in distilled water to remove excessive DMSO and to form aqueous suspensions of PLGA/PVA-coated PSiNPs.

For comparison we have prepared and studied a suspension of uncoated PSi particles by using the powder of as-prepared PSi films mixed with water. Prior to the investigation, the formed suspensions were subjected to ultrasonication for 15 min.

Some parts of the aqueous suspension were used to deposit the nanoparticles on flat surface of c-Si wafer. The dried samples were investigated by means of scanning electron microscopy (FE-SEM, Sirion, FEI Company, Hillsboro, OR, USA) at an acceleration voltage of 10 kV. Additionally, the samples were studied using a Fourier-transform infrared (FTIR) spectrometer (Alpha-P, Bruker Corporation, Billerica, MA, USA) with attenuated total reflectance mode.

The PL spectra of the aqueous suspensions of PSiNPs were measured using a spectrophotometer Perkin Elmer LS-55 (PerkinElmer Inc., Waltham, MA, USA) under continuous wave excitation by a Xe lamp (with excitation wavelength of 350 nm and spectral width of 10 nm).

The PL relaxation transients were detected by R928 photomultiplier tube (Hamamatsu Photonics, Hamamatsu, Shizuoka, Japan) under pulsed laser irradiation by a nitrogen laser (excitation wavelength of 337 nm and pulse duration of 10 ns). The time response of the detection system was better than 1 μs.

The PL quantum yield was measured by comparing the PL intensity and absorption of the samples and a solution of Rhodamine 6 G with the PL quantum yield of about 100%.

## Results and discussion

### Samples characterization

**Figure 1:**
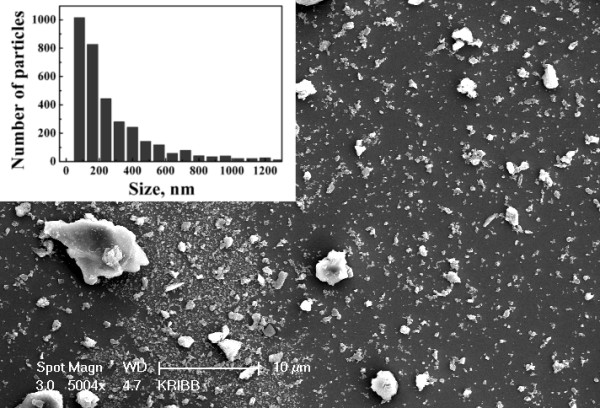
**SEM images of uncoated nanoparticles after ultrasonic treatment.** Inset shows size (diameter) distribution of PSiNPs deposited on c-Si substrate.

Figure 1 shows a typical SEM image of the dried uncoated PSiNPs deposited on c-Si substrate. One can see both relatively big particles with sizes up to 4 μm and smaller ones with diameter less than 200 nm (see size distribution in the inset of Figure 1).

**Figure 2:**
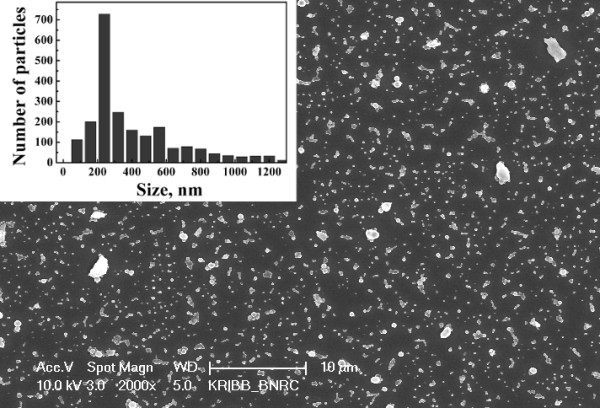
**SEM images of coated nanoparticles after ultrasonic treatment.** Inset shows size (diameter) distribution of PSiNPs.

The polymer coating changes the size distribution of PSiNPs in comparison with uncoated ones. Figure 2 shows that there are no big particles with sizes above 2 μm. The size distribution of the coated PSiNPs has a maximum size at approximately 200 nm (see the inset of Figure 2). The latter value is larger than that for uncoated PSiNPs. The observed modification of the size distribution can be explained by an influence of the PLGA/PVA shell, which stimulates agglomeration of smaller PSiNPs.

**Figure 3:**
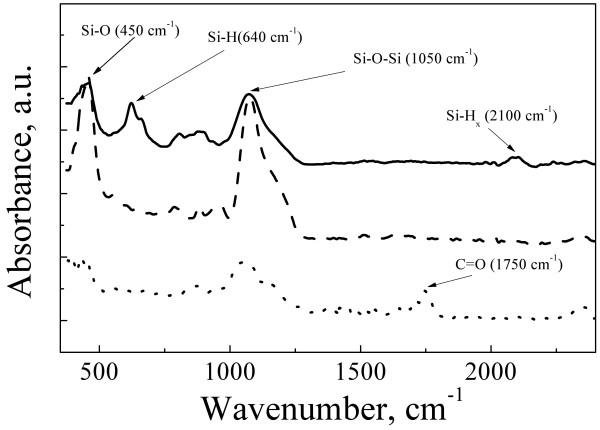
**FTIR absorption spectra of the samples: uncoated sample before storage in water (solid line), uncoated sample after storage in water (dashed line) and PLGA/PVA-coated sample after storage in water (dotted line).** The storage was done in darkness for 2 weeks at room temperature.

The FTIR spectra of the dried samples were measured in order to determine the chemical composition of the PSiNP surfaces (see Figure 3). The uncoated samples exhibit spectra with dominant absorption peaks at 640 cm^-1^ and approximately 2,100 cm^-1^, which correspond to the Si-H wagging and Si-H_*x*_ (*x* = 1,2,3) stretching modes, respectively [25]. Note that the hydrogen-passivated surface is typical for PSi obtained by electrochemical etching in HF solutions [6,24]. Additionally, two peaks at 1,050 cm^−1^ and at 450 cm^−1^ related to the Si-O stretching and rocking modes, respectively, point to the partial oxidation of PSiNP surfaces. The storage for 2 weeks of uncoated PSiNPs in water significantly increased the absorption intensity of Si-O bonds and suppressed its absorption by (Si_3_-)Si-H bonds, which probably transformed into (Si_*x*_O_*y*_-)Si-H bonds. The detection of (Si_*x*_O_*y*_-)Si-H bonds was hampered by their smaller intensity of the absorbance. Dotted line in Figure 3 shows the FTIR spectra of PLGA/PVA-coated PSiNPs. New peak at approximately 1,750 cm^−1^ is related to the C = O bond [26], and it is indicative for PLGA molecules [27]. On the one hand, the comparable intensities of the Si-O peaks for the uncoated and coated samples evidence the limited oxidation of PSiNPs surface during the coating procedure. On the other hand, the absence of (Si_3_-)Si-H bond absorption points to the strong losses of hydrogen from the PSiNP surfaces. This effect can be related to the treatment in DMSO.

### Enhancement of photoluminescence efficiency

**Figure 4:**
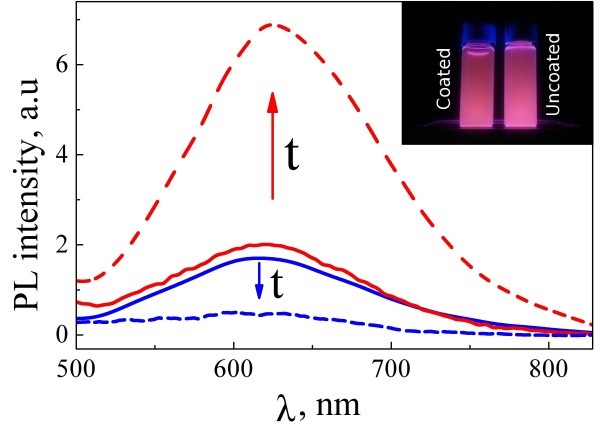
**Examples of PL spectra.** PL spectra of uncoated (blue line) and coated (red line) samples before (solid lines) and after (dashed lines) 4 days storage. Inset shows photograph of aqueous suspensions of coated and uncoated PSiNPs under UV irradiation just after the preparation.

Both suspensions of uncoated and coated PSiNPs just after preparation possessed efficient PL under irradiation with the ultraviolet (UV) light (see inset of Figure 4). The PL intensities of the two samples were close to each other at the beginning of the storage. Initially, the PL quantum yield was estimated to be about 5% for both samples.

**Figure 5:**
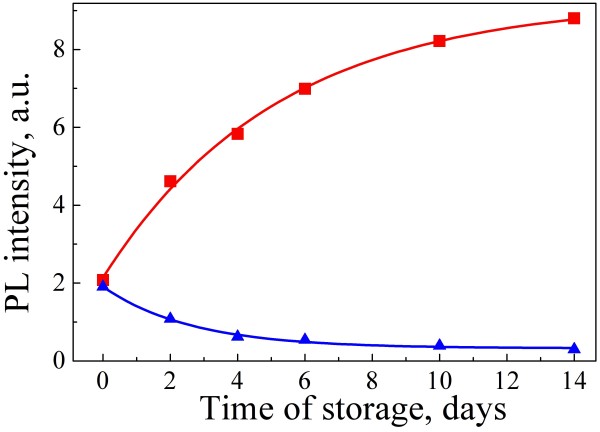
**Time dependences of the integrated PL intensity of the samples: uncoated PSiNPs (blue triangles) and coated PSiNPs (red squares).** Solid lines are results of nonlinear fitting by Equations 1 and 2, respectively.

Stability of the PL intensity of PSiNPs is a key point for bioimaging application. Figure 4 shows PL spectra of the uncoated (blue curves) and coated (red curves) PSiNPs just after formation of suspensions (solid lines) and after 4 days storage (dashed lines). The time dependences of the spectrally integrated PL intensities were measured for both uncoated (squares) and coated (triangles) samples (see Figure 5). The integrated PL intensity of uncoated PSiNPs decreases during storage in water, while the form of the spectrum maintains approximately the same. The PL degradation of PSiNPs can be related to photostimulated reactions with molecular oxygen, which results in the formation of silicon dangling bonds [7,28,29]. In the present work, we intentionally used short illumination time (approximately 1 min) and low intensities (approximately 1 mW/cm^−2^) of the excitation in order to minimize the possible effects of photostimulated reactions. So, the main reason of the PL degradation of PSiNPs is expected to be the formation of Si dangling bonds because of the dissolution of PSiNPs in water [17]. It is known that the dangling bonds are responsible for the nonradiative recombination of charge carriers in nc-Si with the recombination rate inversely proportional to the nc-Si diameter [30]. Since the PL spectral shape does not change along the degradation process, one can assume that the dissolution instantly quenches nc-Si. The resulted PL spectrum of PSiNPs is a superposition of the contribution of nc-Si which are not subjected to the dissolution.

**Figure 6:**
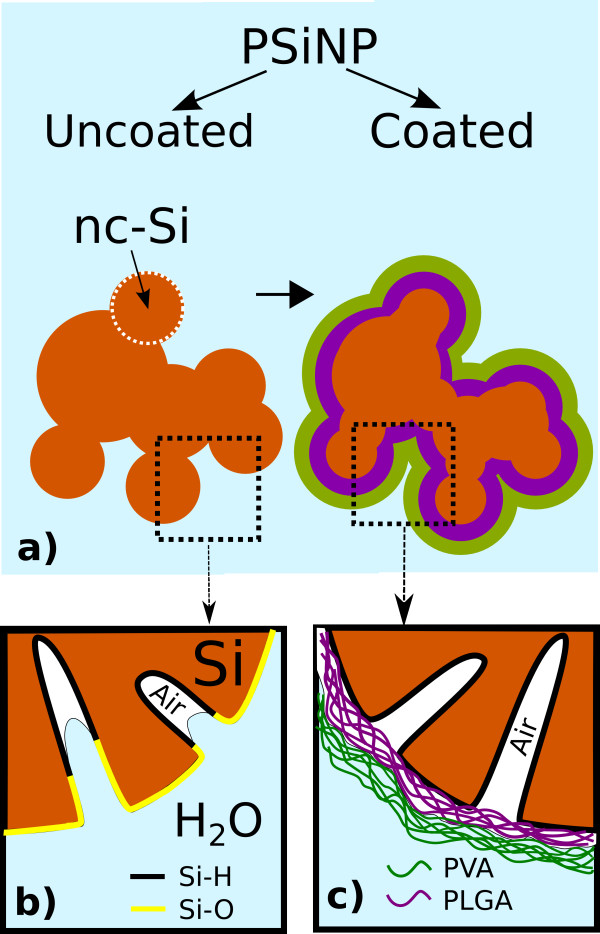
**Sketch of the samples.** (**a**) A schematic view of the porous Si nanoparticle (PSiNP) coating process where brown color corresponds to silicon, violet to PLGA, green to PVA and light blue to water. Note that every PSiNP includes several smaller nanocrystals (nc-Si). (**b**) Magnification of the uncoated PSiNP surface contiguous to liquid water. Hydrophobic surface mostly consisted of Si-H bonds was colored black and hydrophilic one mostly consisted of Si-O bonds was colored yellow. (**c**) Magnification of the coated PSiNP surface. Violet and green lines correspond to PLGA and PVA molecules. Note that liquid water is not in direct contact with the silicon surface of PSiNPs due to the hydrophobic properties of PLGA.

Liquid water can penetrate into oxidized parts of the pores of uncoated samples during the storage (sketched in Figure 6 b). Water turns the surface of uncoated PSiNPs from hydrophobic to hydrophilic. New areas of hydrophilic surface allow further water penetration into the pores. This penetration results in the formation of new dangling bonds on the nc-Si surface.

A tendency is opposite in the case of coated PSiNP (see squares in Figure 5). The PL intensity increases due to the passivation of non-radiative defects by water molecules in a gas phase. Water vapors are known to perform good passivation of the dangling bonds [29]. We suppose that coated PSiNPs are protected from the interaction with the liquid phase of water by hydrophobic PLGA layer. On one hand, the enhancement of PL takes place due to the interaction with water vapors present in pores of SiNPs (see Figure 6 c). On the other hand, the PVA coating provides the hydrophilic properties of PSiNPs. The PL properties of coated PSiNPs become stable after 1 month of storage due to the saturation of described process.

The time dependence of the PL intensity of uncoated PSiNPs is well fitted by the following formulae (plotted by blue curve in Figure 5):

(1) $$I\left(t\right)=I_{0}\exp\left(-t/t_{d}\right)+I_{\mathrm{res}}$$

where is *I* the initial PL intensity, *t* is the characteristic time of PL degradation, *I*_res_ is the residual PL intensity. The time dependence of the PL intensity of coated PSiNPs is well fitted by the following function (plotted by red curve in Figure 5):

(2) $$I\left(t\right)=I_{0}+I_{\mathrm{rec}}\left[1-\exp\left(-t/t_{e}\right)\right]\text{,}$$

where *t*_e_ is the characteristic time of PL enhancement, *I*_rec_ is the PL intensity of nc-Si with defects, which are able to be passivated during storage in water.

By fitting the experimental data with Equations 1 and 2, one can obtain the values of *t*_d_ and *t*_e_ to be about 2.5 and 5 days, respectively. The obtained *I*_res_ is about 20 times smaller than the final PL intensity of coated PSiNPs (*I*_0_ *+ I*_rec_). According to our measurements, the PL quantum yield of coated PSiNPs increased from about 5% to 20% during the storage and it was stable for the next month and afterwards.

### Photoluminescence transients

**Figure 7:**
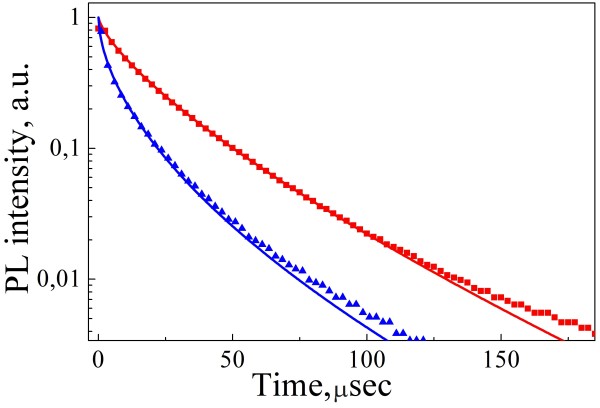
**Transients of the PL relaxation.** Transients of coated PSiNPs are depicted by red squares and transients of uncoated PSiNPs are depicted by blue triangles (detection wavelength is 600 nm). Lines are results of fitting by Equation 5.

The PL transients provide additional information about the mechanisms of the charge carrier recombination. Figure 7 shows the PL transients of both uncoated (red triangles) and coated (blue squares) PSiNPs. The PL was detected at the maximum position of the corresponding spectra (600 nm). Supposing only the radiative way of charge carrier recombination the PL transients would follow the monoexponential law as follows:

(3) $$I\left(t\right)=I\left(0\right)\exp\left(-t/\tau_{r}\right)\text{,}$$

where *I*(0) is the PL intensity just after the pulsed laser excitation; *τ*_r_ is the radiative lifetime of excitons in nc-Si. Wide distribution of the defects *f(τ)* with recombination lifetimes, *τ* , leads to the following law of PL relaxation [31] as follows:

(4) $$I\left(t\right)=I\left(0\right)\int_{0}^{+\infty }f\left(\tau \right)\exp\left(-t/\tau \right)d\tau \text{.}$$

Equation 4 was used to describe the PL transients of self-trapped excitons confined in nc-Si [31] in the following equation:

(5) $$I\left(t\right)=I\left(0\right)\exp\left[-{\left(t/\tau_{0}\right)}^{\beta }\right]\text{,}$$

where *τ*_0_ is the mean decay time and *β* is a parameter related to the dispersion of *f(*τ*)*.

The fitting of the PL transients by Figure 5 gives *τ*_0_ = 5.1 μs, *β* = 0.57 for uncoated PSiNPs (blue line in Figure 7) and *τ*_0_ = 16 μs, *β* = 0.73 for coated PSiNPs (red line in Figure 7). Since the PL lifetime is significantly longer for the coated PSiNPs than for uncoated ones, it evidences the lower defect concentration for nc-Si in coated PSiNPs. The higher value of *β* for the coated samples points to a smaller energy dispersion of the defect states. Note that the obtained *τ*_0_ and *β* values for the coated PSiNPs are close to the corresponding values for highly luminescent nc-Si in SiO_2_ matrix formed by high temperature annealing of SiO/SiO_2_ structures [32]. This fact confirms the suggestion about perfect passivation of the nonradiative defects in the coated PSiNPs.

## Conclusions

We have demonstrated the significant enhancement of the photoluminescence efficiency of aqueous suspensions of porous silicon nanoparticles covered by PLGA/PVA. This polymer coating was suggested to prevent silicon nanocrystals in porous silicon from dissolution in water due to the presence of hydrophobic PLGA layer. At the same time, the passivation of the defects on Si nanocrystal surfaces via interaction with water vapors was achieved. The passivation led to the continuous increase of the photoluminescence intensity for 2 weeks. Since the polymer-coated nanoparticles demonstrate high quantum efficiency of photoluminescence (up to 20%) and stable luminescent properties after 1 month storage in water, they are promising for bioimaging applications both *in vitro* and *in vivo.* Another advantages of both uncoated and PLGA/PVA-coated porous silicon nanoparticles as labels for bioimaging are their high bioresorbability and biocompatibility.

## Competing interests

The authors declare that they have no competing interests.

## Authors' contributions

MBG performed fabrication of the PSi samples, measurements of kinetics, data analysis and wrote the text of the article. AYK participated in fabrication of the PSi samples, carried out most part of the experiments, and also wrote some parts of the article. JJ, HL, and BHC contributed in PLGA/PVA coating and other measurement of structural properties. VYT and LAO performed the general data analysis and discussion of the obtained data. All authors read and approved the final manuscript.
